# Self-reflection, sense of agency, and underlying neural correlates: A pilot study

**DOI:** 10.1371/journal.pone.0335276

**Published:** 2025-12-26

**Authors:** Kurusetti Vinay Gupta, Jyotiranjan Beuria, Lokeswara Kumar Vijanapalli, Amit Sethi, Laxmidhar Behera

**Affiliations:** 1 Department of Electrical Engineering, Indian Institute of Technology Kanpur, Kanpur, India; 2 Indian Knowledge System and Mental Health Applications Center, Indian Institute of Technology Mandi, Mandi, India; 3 Indian Knowledge System Research Center, Institute for Science and Spirituality Delhi, Delhi, India; 4 Department of Occupational and Recreational Therapies, University of Utah, Salt Lake City, Utah, United States of America; Museo Storico della Fisica e Centro Studi e Ricerche Enrico Fermi, ITALY

## Abstract

The sense of agency refers to the feeling of controlling one’s actions to influence the outside world. The association between an individual’s sense of agency and their psychological or behavioral characteristics has been extensively studied in recent years. However, the direct impact of self-reflection, a key strategy for modulating one’s cognitive state, on implicit measures of agency remains largely unexplored. We employed a between-subjects experimental design, to investigate the effect of two types of self-reflection - self-centered and selfless upon the implicit sense of agency. Additionally, we explored neural activity during self-reflection to see if there are any correlations with the sense of agency. Agency was measured using intentional time binding experiments, assessing the ability to perceive event sequences over time. Results indicated that self-centered reflection enhances time binding and agency, while selfless reflection has the opposite effect. Traditional spectral density measures and topological data analysis revealed distinct neural patterns for each type of reflection. Specifically, selfless reflection showed increased Hodge spectral entropy and persistent entropy compared to self-centered and control groups, indicating greater topological complexity in EEG time series. A significant negative correlation between second-order Hodge spectral entropy and time binding effect was observed. The study provided initial evidence that topological EEG features could serve as potential neural markers of the sense of agency modulated through self-reflection.

## Introduction

Sense of agency refers to the conscious experience of being in control of one’s actions [1]. Thus, its pervasive nature in conscious awareness of one’s interaction with the external world has attracted the attention of psychologists, neuroscientists, and philosophers. Despite being a key factor interlinked with our minimal self-hood [2], the experience associated with the feeling of agency over one’s actions is not as salient as one would expect. Thus, it remains challenging to study such a ‘phenomenally thin’ [3] observation.

In the last two decades, several attempts have been made to measure the sense of agency, which have been broadly categorized into two groups of measurement, i.e., explicit and implicit measures [4]. The explicit measures of agency rely on subjective self-reports and, thus, suffer from consistent cognitive bias in terms of misattribution of agency, especially for the actions with positive outcomes [5,6]. On the other hand, implicit measure attempts to quantify the feeling of agency without explicitly making one think over agency over the performed action. Following the seminal work of Patrick Haggard and colleagues [7], distortion in time perception in an ‘intentional binding’ setup has been reported to be linked with the implicit measure of sense of agency. The perceived compression of the time interval between a voluntary action and its perceived external sensory outcome is referred to as the intentional binding effect [7].

Many researchers have explored the connection between intentional binding and psychological or behavioral traits. Recent studies have shown that interoception, which is the awareness of the body’s internal physiological state, like the systole or diastole phase in the cardiac cycle, affects the sense of agency [8,9].

Studies have reported about the so-called ‘social sense of agency’ where one’s activities trigger the reactions of others, one’s actions together modulate their reactions, or their simple social presence affects one’s sense of agency [10]. In a work by Yoshie and Haggard [11], social-emotional modulation of sense of agency has been reported in which the authors identified a mechanism whereby routinely negative action outcomes could influence sense of agency. Some recent studies have also discussed the impact of social, affective, and other kinds of non-motoric cues in modulating the sense of agency [12–14]. The researchers in [12] found that emotions like fear or anger can affect the subjective sense of control over an action outcome, even though the actual causal relationship between action and outcome remains unchanged. According to [15], the emotional content of an intention is significant, indicating that implicit bias is heightened when we express a negative intention. Intentional binding has been influenced by beliefs in free will [16], obsessive-compulsive tendencies [17], and exposure to emotionally positive images [18]. It has also been affected by low or vulnerable-type narcissism [19,20] and the recall of episodic memories in which individuals felt a sense of power over others [21], as well as the activation of depressing memories [22].

It is worth mentioning that the impact of interventions such as self-reflection and meditation, which directly affect different aspects of one’s sense of self, on the implicit measure of sense of agency has not received much attention. Recently, Lush et al. [23] reported that mindfulness meditators exhibited stronger intentional binding (stronger compression between action and outcome) compared to subjects easily hypnotizable due to higher meta-cognitive access to one’s intentions [23,24]. On the contrary, Chiarella et al. [25] reported weakening intentional time binding for mindfulness meditators due to less pronounced attachment to self [25]. Thus, an absence of clear consensus over how self-reflection and contemplation affect the sense of agency calls for more studies in this direction. Moreover, Silvanto and Nagai [26] have recently proposed a theoretical framework emphasizing the role of interoception and the insula-anterior cingulate cortex network in integrating bodily signals with sensory and cognitive information, which is critical for vivid mental imagery and a robust sense of agency. Therefore, it is important to investigate the neural correlates of self-reflection and meditation in relation to implicit measures of agency. This also underscores the need to study how electroencephalogram (EEG)-based neural markers of interventions such as self-reflection relate to the implicit sense of agency.

In this study, we aimed to explore the effects of self-reflection upon sense of agency and the neural underpinnings, by examining two distinct modes: self-centered reflection and self-less reflection on life events.

The first mode, termed “self-centeredness", involved the self as a central reference point in various psychological activities, marked by biased self-interest and an inflated sense of importance. In contrast, the second aspect, termed “selflessness", was characterized by diminished self-centeredness and a reduced emphasis on the self’s significance. This latter mode of functioning was associated with altruism, kindness, empathy, and a weak distinction between self and others [27].

Thus, to gain a comprehensive understanding of these cognitive states, we examined traditional EEG features such as band powers and novel topological data analysis (TDA) [28,29] based measures like Hodge spectral entropy [30], persistent entropy, and persistent amplitude. We aimed to identify neural correlates associated with two types of self-reflection. Topological features of EEG time series have been successful in uncovering meaningful insights into cognitive processes, emotional states, and neurological disorders [30–34]. This approach transforms EEG time series data from every single electrode into a point cloud in a higher-dimensional space and extracts temporal topological features at various scales of filtration.

Our study found that self-centered reflection (SCR) on life events enhanced the overall sense of agency through increased judgment error in time perception, also known as total time binding (TTB), unlike a control group (CTR) without self-reflective activity. On the other hand, selfless reflection (SLR) reduced TTB, decreasing the sense of agency compared to the CTR group. The topological signatures and band power features of EEG signals exhibited significant differences between self-centered and selfless reflections. Our findings revealed a statistically significant negative correlation between the sense of agency and Hodge spectral entropy, which denoted the amount of topological information needed to describe the temporal connectivity patterns of a single EEG channel’s time series. Thus, a negative correlation indicated that a stronger sense of agency might be associated with less complex temporal connections of the neural processes. This could be a potential biomarker associated with different reflective mental states. We also explored another measure of temporal complexity known as multiscale entropy (MSE). We found that the MSE for self-centered reflection was lower than the selfless and control groups. Higher MSE values indicated greater signal complexity, which varies significantly in different neurological conditions [35,36].

The organization of this manuscript is as follows. We first delineate the experimental framework. Next, we briefly introduce the essential aspects of TDA and the definitions of different topological measures. We then present our findings and associated discussions. Finally, we conclude with a summary of our work.

## Experimental framework

### Hypothesis

The primary objective of this study was to investigate whether the type of self-reflection - SCR, SLR, or CTR would affect the implicit sense of agency. The primary hypothesis was that self-reflection (SCR,SLR) would significantly change the implicit sense of agency (as measured by TTB) compared to CTR. The secondary objective was to identify the neural correlates of sense of agency. We hypothesized that SCR and SLR would show distinct EEG signatures, both in spectral band powers and topological features compared to CTR. Additionally, we examined the association between the topological EEG features and sense of agency in various types of self-reflection (SCR, SLR and CTR). We hypothesized that the Hodge spectral entropy [30], which is a topological EEG feature would significantly correlate with the magnitude of TTB. These hypotheses are inspired by recent findings showing that non-motoric factors such as emotional content, self-related processes, and interoceptive awareness can modulate the sense of agency [12,24,26], suggesting a compelling rationale to explore how self-reflection—an introspective, self-referential process might influence both behavioral and neural measures of agency.

### Participants

Ninety-two participants (47 males, 45 females), aged between 16 and 30 years, with an average age of 21.5 years (standard deviation = 4.0) were recruited from an online notification between February 2023 and December 2023. Participants received remuneration worth 200 Indian Rupees for participation. Using Wilcoxon signed rank for paired values with an alpha of 0.05, the sample size was determined by a power analysis using G*Power [37], which showed a minimum sample size of 33 participants was required to identify a medium effect (Cohen’s d = 0.6) with 95% power. Participants were eligible if they were right-handed, had normal or corrected-to-normal vision, and reported no history of neurological or psychiatric disorders. Anyone who did not meet all of these criteria was excluded from the study. Before the experimental session, written informed consent was obtained from all subjects and the parents and/or legal guardians of one minor involved in this study. This study was approved by the Institute Ethical Committee (Human) of IIT Mandi (IITM/IEC(H)/2023/VD/P2).

### EEG recording

We recorded EEG signals using a commercially available headband-based EEG system, Muse-S (InteraXon Inc.) [38]. The system comprised four channels, where two dry electrodes were positioned on the forehead at AF7 and AF8 and the two behind the ears at TP9 and TP10 with a reference electrode Fpz. We employed this commercially available system known for its user-friendly design and portability. While it offered a limited number of channels compared to more advanced research-grade EEG systems, its accessibility and real-time feedback capabilities made it an effective tool for this pilot, between-subjects experimental study.

The sampling frequency of the signals recorded from the device is 256 Hz. The EEG data were streamed using python library muselsl [39] and recorded using custom code developed based on pylsl inlet stream module. This pipeline was validated using BrainFlow, a standard Python-based EEG streaming and recording package. The stimulus was presented using PsychoPy v2022.2.5 [40].

### Experiment design

We employed a between-subject experimental design. During the experiment, participants were comfortably seated in a quiet room, 50 cm from a MacBook Pro (13.3-inch screen) with a screen resolution of 2560 x 1600 pixels and a refresh rate of 60 Hz, equipped with an Apple M2 processor. Participants were randomly assigned to one of three groups:

- SCR (16 males, 15 females) for 240 seconds,
- SLR (16 males, 15 females) for 240 seconds,
- CTR (15 males, 15 females) for 240 seconds.

Participants in the SCR group were asked to reflect on eight questions (30 seconds each) related to their past and future, focusing on personal successes and failures. These questions were as follows:

- Visualize the last time someone praised you a lot.
- Visualize the last time someone rebuked you harshly.
- Reflect on occasions when your hard efforts brought you great success.
- Reflect on occasions when you failed despite putting in significant effort.
- How do you perceive yourself when you stand before a mirror now?
- How do you imagine you will look when you stand before a mirror in 25 years?
- Contemplate your biggest current worry.
- Contemplate the anxieties you have about your future.

These questions were inspired by previous studies [41] investigating the effects of autobiographical recall on cognitive performance. Participants in the SLR group were asked to reflect on eight questions (30 seconds each) related to acts of selflessness. These questions were as follows:

- Think of a time when you felt grateful for your body as a gift from God.
- Recall a moment when you helped someone without expecting anything in return.
- Think of a situation when you shared something important with someone in need.
- Remember when you stayed calm in a difficult situation, trusting God’s plan.
- Recall a time when you prayed for guidance to act according to God’s will.
- Think of a time when you let go of your ego and embraced humility.
- Visualize contributing to others’ well-being in the future.
- Envision yourself sharing joy and wisdom, not for credit, but to enrich the lives of others.

These questions were originally part of a mantra-based CBT study [42] to invoke selflessness. Prior to formal data collection, both SCR and SLR prompt sets were pilot-tested on 12 volunteers representative of the target population; their structured feedback led to minor wording refinements that improved clarity, emotional resonance, and ecological validity.

The CTR underwent no specific contemplation and sat silently for 240 seconds. Appropriate event markers were recorded during different experiment phases using the Pylsl framework [43]. This passive resting-state condition served as a baseline for comparison with the active reflection conditions.

#### Intentional time binding.

**Fig 1:**
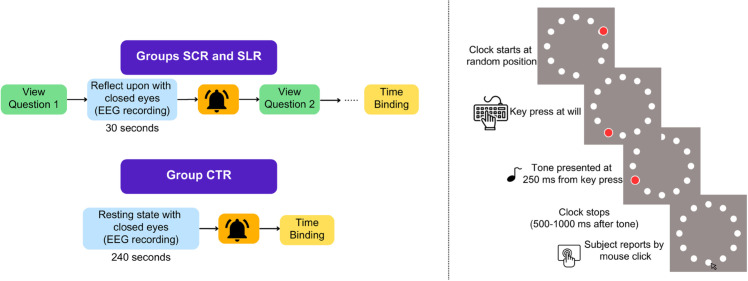
(Left) Overall pipeline of the experiment for the three groups. A beep sound follows every reflection to indicate to open the eyes for the next task. An intentional time binding experiment is carried out for each group at the end. (Right) Schematic timeline of the intentional time binding experiment.

Following the reflections above, each group underwent a cognitive experiment on intentional time binding to measure the judgment error in time perception. This cognitive task was popularised by Haggard et al. [7] to study the implicit measure of sense of agency. The task had four randomized conditions, i.e., action-only, tone-only, and two action-tone. Each condition included 20 trials, for a total of 80 trials. The block diagram for the experiment is given in Fig 1. The total duration of the task was around 15 minutes.

In all the trials, participants were confronted with a clock featuring a red dot rotating along the edge. In the action-only condition, they pressed any key at will while the red dot rotated. After their key press, the red dot stopped its rotation after a random, short duration. After the red dot stopped rotating, participants clicked on the clock face at the location where they believed the red dot was positioned at the moment they pressed the key.

In the tone-only condition, participants waited for an audio tone. After hearing the tone, the red dot stopped rotating after a brief, random delay. Participants then clicked on the clock face at the location where they believed the red dot was positioned at the moment of the tone. These two conditions served as the baseline.

In action-tone conditions, the participants initiated actions by pressing a key at their discretion, followed by a 250-millisecond delay before an audio tone sounded. This condition had two variations: in one, participants clicked on the clock face where they thought a red dot was at the time of the key press; in the other, they clicked where they believed the red dot was at the time of the audio tone.

Participants also filled out a questionnaire that asked them to mark the number of erroneous markings they believed they made in each phase. The markings were based on their confidence in their own judgment about the red dot’s location. At the end of the experiment, perceived mental load and feedback about the overall experiment procedure were rated on a scale of 1 to 5. This information was used to assess if any participants should be excluded from the analysis due to excessively negative feedback, which might indicate a lack of engagement or difficulty in understanding the task. The responses from two participants were excluded from the time binding analysis on the basis of subjective reports that they had fallen asleep during the time binding task.

### EEG pre-processing

The recorded EEG signals were filtered by a linear finite impulse response filter of fourth order with a high-pass filter at 0.1 Hz and a low-pass filter at 45 Hz using a popular Python library, MNE. A notch filter of 50 Hz was applied to filter power line noise. The filtered data were decomposed into maximally independent components using FastICA method. Although ICA is not recommended for low-density EEG systems like Muse for eye-blink removal, we employed it primarily for muscle-artifact removal. Eye-blink removal was not a necessity since the reflections were associated with closed eyes. MNE also provided functions to automatically find the independent component that represented muscle artifacts that were produced on account of postural maintenance and were cleaned using MNE. Muscle-related components tend to have a positive log-log spectral slope between 7 Hz and 45 Hz and tend to have a single focal point (not spread out). We compared each ICA component to a typical muscle-related one. We considered it a muscle artifact if it was similar in terms of slope, focal point, and smoothness. For two subjects of each group, the muscle artifact components were so prominent that we had to reject these subjects for EEG-based analysis. Since TDA was computationally demanding for large time series, we downsampled the EEG signals by a factor of 4 to obtain signals with a sampling rate of 64 Hz for TDA computations only.

The analysis involved using uniform durations of preprocessed EEG signals for all interventions. Each question in the SCR and SLR intervention lasted for 30 seconds. The EEG features corresponding to all eight questions were averaged to generate the EEG representation for the respective intervention, resulting in a duration of 30 seconds. We used the last 25 seconds of these EEG time series for subsequent analysis. For the CTR group, the analysis focused on the central 25 seconds of the 240-second session.

Because of the similar range and trends of TP9 and TP10, we pooled them by averaging them to form a TP channel. Similarly, AF7 and AF8 were pooled to form an AF channel. While the electrode placement deviated from traditional EEG setups, the system was validated in numerous research studies on meditation [44–46].

## Theoretical framework

### Topological features

The EEG time series data is embedded as a point cloud using Takens’s embedding theorem [48]. According to Takens’s embedding Theorem, we can construct vectors in a higher-dimensional space by taking successive points from the time series. Suppose we have a time series $\left(x(t_{1}),x(t_{2}),\ldots ,x(t_{n})\right)$. We obtain the resulting vectors in the reconstructed *m*-dimensional space:

$$\left(x(t_{1}),\;x(t_{1}+\tau ),\;x(t_{1}+2\tau ),\;\ldots ,\;x(t_{1}+(m-1)\tau )),\;(x(t_{2}),\;x(t_{2}+\tau ),\;x(t_{2}+2\tau ),\;\ldots ,\;x(t_{2}+(m-1)\tau )),\;\vdots \;(x(t_{n-m+1}),\;x(t_{n-m+1}+\tau ),\;\ldots ,\;x(t_{n-m+1}+(m-1)\tau )\right)$$ (1)

Here, *m* is the embedding dimension, and τ is the time delay. If the system is periodic, the point cloud might resemble a loop or closed curve. In the case of chaotic dynamics, the point cloud might exhibit a fractal-like, intricate structure.

**Fig 2:**
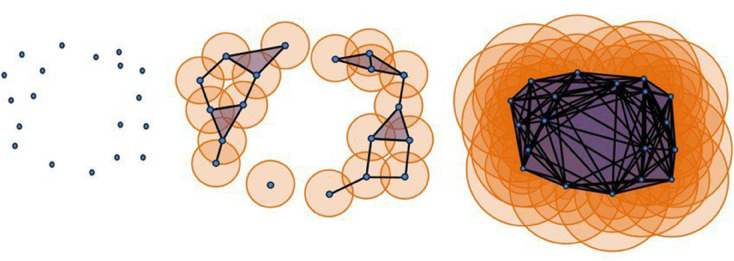
Generating Vietoris-Rips (VR) Complexes from Point Cloud Data: Each point in the point cloud is constructed from an EEG time series using Takens’s embedding theorem. Each point is enveloped by a growing sphere, which simultaneously and uniformly expands, forming k-simplices [47].

In TDA, the primary component is constructing a simplicial complex. It consists of simplices such as points (0-simplex), edges (1-simplex), and triangles (2-simplex) etc. For a point cloud $P=\{p_{1},\ldots ,p_{N}\}$, we construct a simplicial complex using the Vietoris Rips (VR) algorithm (see Fig 2). A k-simplex represents a collection of (k + 1) points in *P* that are sufficiently close to each other, according to the specified distance threshold *r*. Given *r* > 0, we thus define the simplicial complex *K*_*r*_ by considering all simplices $\sigma =\{x_{1},\ldots ,x_{l}\}\subseteq P$ such that $\|x_{i}\;-\;x_{j}{\|}_{2}<r$ for all $x_{i},x_{j}$ in σ. In the VR complex, *r* is the filtration parameter.

One of the key variables in this analysis [30] is the so-called Betti numbers ($\beta_{k}$) corresponding to *k*^*th*^ homology dimension. $\beta_{k}$ counts the number of *k*-dimensional holes in the simplicial complex. $\beta_{0}$ counts the number of connected components, $\beta_{1}$, the number of 1-dimensional holes or circles, and $\beta_{2}$, the 2-dimensional holes or voids. Next, we present a few important topological features used in this work.

#### Persistent homology features.

Persistent homology accounts for the topological features that remain persistent across different values of filtration parameter *r*. They are as follows.

- Betti curve β(.): The Betti curve, represented as $\beta (D_{k}):\mathbb{R}\to \mathbb{N}$, is a function that counts the multiplicity of points in the $k^{\text{th}}$ homology group associated with a filtration *D*_*k*_ at a specific filtration parameter *r* = *s*. This counting is constrained by the condition $b_{i}\leq s\leq d_{i}$, where *b*_*i*_ denotes the *r* at which the feature appeared, also termed as ‘birth’ of the $i^{\text{th}}$ topological feature and *d*_*i*_ indicates its ‘death’ or disappearance of the feature. Consequently, the Betti curve provides insights into the persistence of topological features as the filtration parameter varies.
- Persistence entropy ($S_{k}^{pe}$): The persistence entropy quantifies the distribution of points in a persistence diagram (collection of persistence intervals *D*_*k*_), reflecting the complexity of topological features. Let $D_{k}=\{(b_{i},d_{i})\}$ denote the set of birth–death pairs for the $k^{\text{th}}$ order homology group, with $d_{i}<\infty$. The $k^{\text{th}}$ order persistence entropy is defined as
  $$S_{k}^{pe}=-\sum_{i=1}^{\left|D_{k}\right|}p_{i}\log(p_{i}),$$ (2)
  where
  $$p_{i}=\frac{d_{i}-b_{i}}{L_{D_{k}}},\;L_{D_{k}}=\sum_{i=1}^{\left|D_{k}\right|}(d_{i}-b_{i}).$$
- Persistent amplitude (*A*_*k*_): The persistent amplitude for the $k^{\text{th}}$ order homology group is defined on *D*_*k*_, the set of persistent $\left(b_{i},d_{i}\right)$ pairs, as a function $A_{k}:D_{k}\to \mathbb{R}$, for which there exists a vectorization $\Phi :D_{k}\to V$, where *V* is a normed space. The relationship between the amplitude and the vectorization is given by $A_{k}(x)=\|\Phi (x)\|$ for all $x\in D_{k}$. For norm computations, we utilize the Wasserstein metric.

#### Hodge spectral entropy.

In information theory, entropy is a metric for the degree of randomness or uncertainty in a system. In TDA, one finds the eigenvalues of the so-called Hodge Laplacian *L*_*[k]*_ [30,49], a higher-dimensional analog of the graph Laplacian. Hodge spectral entropy $S_{n}^{hs}$ is expressed as follows:

$$S_{n}^{hs}=\alpha \langle \lambda_{n}\rangle +\log(Z_{n}),\;Z_{n}=\text{Tr}\;\left(e^{-\alpha L_{\left[n\right]}}\right),\;\langle \lambda_{n}\rangle =\frac{\sum_{i=1}^{N_{n}}e^{-\alpha \lambda_{i}(L_{\left[n\right]})}\;\lambda_{i}(L_{\left[n\right]})}{Z_{n}},$$ (3)

where $\lambda_{i}(L_{\left[n\right]})$ denotes the $i^{\text{th}}$ eigenvalue of *L*_*[n]*_, α is the damping factor, and *N*_*n*_ is the total number of eigenvalues of *L*_*[n]*_. We used the gudhi [50] and hodgelaplacians [51] libraries to compute Hodge spectral entropy. Higher Hodge spectral entropy implies richer topological information content.

### Conventional EEG features

#### Relative band power.

The power spectral density (PSD) is computed using Welch’s method with MNE. We estimate the relative band powers for each intervention in the six bands - delta (0.5 Hz, 5.0 Hz), theta (5.0 Hz, 8.0 Hz), alpha (8.0 Hz, 13.0 Hz), sigma (13.0 Hz, 16.0 Hz), beta (16.0, 30.0 Hz), gamma (30.0 Hz, 45.0 Hz) [52] by taking an average of PSD in each of these bands and dividing with the summation of averages over all the bands.

#### Multiscale entropy (MSE).

MSE facilitates the analysis of signal complexity across various timescales. When dealing with a one-dimensional discrete time series, $\left\{x_{1},x_{2},\ldots ,x_{n}\right\}$, we generate successive coarse-grained time series, represented as $\left\{y^{\left(\tau \right)}\right\}$, corresponding to the scale factor, τ. The original times series is divided into non-overlapping windows of length τ, and subsequently, data points within each window are averaged. In a more general sense, each element of a coarse-grained time series is computed using the equation

$$y_{j}^{\left(\tau \right)}=\frac{1}{\tau }\sum_{i=(j-1)\tau +1}^{j\tau }x_{i},\;1\leq j\leq N/\tau$$ (4)

For the scale factor of one, the time series remains identical to the original. The length of each coarse-grained time series is equivalent to the original time series divided by the scale factor τ. Subsequently, the sample entropy (SampEn) measure is computed for each coarse-grained time series, constituting what we refer to as multiscale entropy ($S_{\text{MS}}$) analysis.

The SampEn for a time series $X=\{x_{1},x_{2},\ldots ,x_{N}\}$ is calculated using a defined pattern length *m* and a tolerance level *r*. The formula is as follows:

$$\text{SampEn}(m,r)=-\ln\left[\frac{A_{r}^{m+1}}{A_{r}^{m}}\right]$$ (5)

Where $A_{r}^{m}$ is the number of sequences of length *m* that are similar, i.e., with a maximum difference between any two corresponding points less than or equal to *r*.

$S_{\text{MS}}$ methodology provides a means to explore the complexity and regularity of data across multiple scales or levels of detail. We have used pyEntropy [53] library for $S_{\text{MS}}$ computation.

## Results

### Effect on time binding

There was an increase in TTB during the intentional binding task in the SCR group compared to the SLR and CTR groups (see Table 1). It indicates that participants reported a larger difference between when they perceived an action or event occurred and when it actually occurred, suggesting a stronger perception of the event being closely related to their intentional actions or mental states after the SCR compared to the other conditions (SLR and CTR).

**Table 1:** Time binding results (in ms). Action/Tone Binding quantifies the difference between baseline and agency-related errors in action and tone perception. Baseline errors are the average time discrepancies between reported and actual actions/tone over 20 trials in action-only or tone-only tasks. Agency-related errors measure the corresponding discrepancies during action-tone tasks. Total time binding reflects the difference between action and tone bindings. Values are presented as mean (SD) across subjects in each group

Measure	Group SCR	Group SLR	Group CTR
Action	-40±84	25±85	6±65
Tone	49±72	39±66	29±65
Total	123±75	51±41	82±54

The three binding effects - action, tone, and total binding were tested for normality for the three groups. Upon the Shapiro-Wilk test for normality of distributions, we found that the Tone Binding associated with Group SCR (p=0.9) and Group SLR (p=0.7) followed normal distributions. Also, the Action Binding effect for Group CTR (p=0.2) was normally distributed. The remaining data were not normally distributed. Thus, Mann Whitney U-test (non-parametric test) was used to compare the means of TTB (see Table 2).

**Table 2:** Omnibus Kruskal–Wallis test and Mann–Whitney post-hoc *p*-values for TTB. Effect-size magnitude is interpreted using conventional cut-offs: small (.01–.06), medium (.06–.14), large (≥.14)

Statistical test	*SCR* *> SLR*	*SLR* *< CTR*	*SCR* *> CTR*
Kruskal–Wallis (omnibus)	**$p=2\times 10^{-6}$, $\epsilon^{2}=0.34$ (large)**		
TTB (Mann–Whitney *U*)	**0.00001**	**0.007**	**0.012**

The choice of single-tailed hypothesis was made in order to validate the hypothesis central to the study that SCR intervention enhances agency while SLR intervention reduces agency compared to the CTR condition. The mean (SD) for Action Binding after the SCR intervention was -40 (84) ms, following the SLR intervention was 25 (85) ms, and after the CTR condition was 6 (65) ms. In contrast, Tone Binding exhibited mean (SD) values of 49 (72) ms after the SCR intervention, 39 (66) ms following the SLR intervention, and 29 (65) ms after the CTR condition. No significant differences were observed between the groups for both action and tone binding based on the Mann-Whitney *U* test.

The mean (SD) for TTB in the SCR group was 123 (75) ms, in the SLR group was 51 (41) ms, and in the CTR group was 82 (54) ms. Statistical analysis revealed that TTB associated with the SCR intervention was significantly higher than the SLR intervention (p(R>M)=0.00001), the SLR intervention was significantly lower than the CTR condition (p(M<C)=0.007), and the SCR intervention was significantly higher than the CTR condition (p(R>C)=0.02). The omnibus Kruskal–Wallis test confirmed a significant group difference ($p=2\times 10^{-6}$), with a large effect size ($\varepsilon^{2}=0.34$).

**Fig 3:**
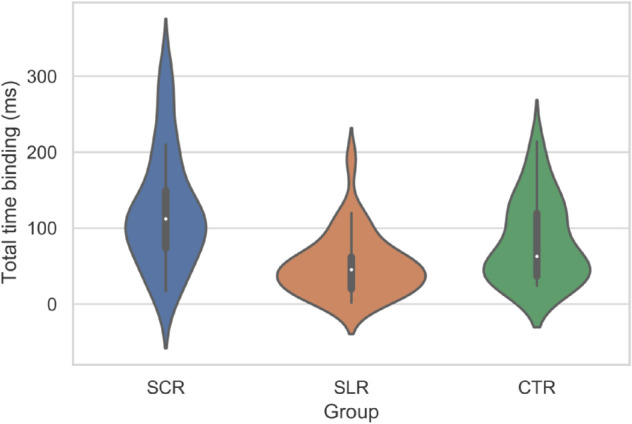
Violin plot representing the distribution of total time binding (in ms) for different groups. Group SCR – Self-Centered Reflection, Group SLR - Selfless Reflection, Group CTR – Control.

Also, the violin plots representing the distribution of TTB are shown in Fig 3. Differences in shape suggest variations in the distributions for the three groups. The medians showed a similar trend discussed above, confirming the hypothesis that the TTB of SCR is greater than CTR, which is greater than SLR. A broader violin shape for the SLR group centered around the median suggested significant data dispersion, particularly clustered around a relatively low median value.

### Effect on relative band powers

**Fig 4:**
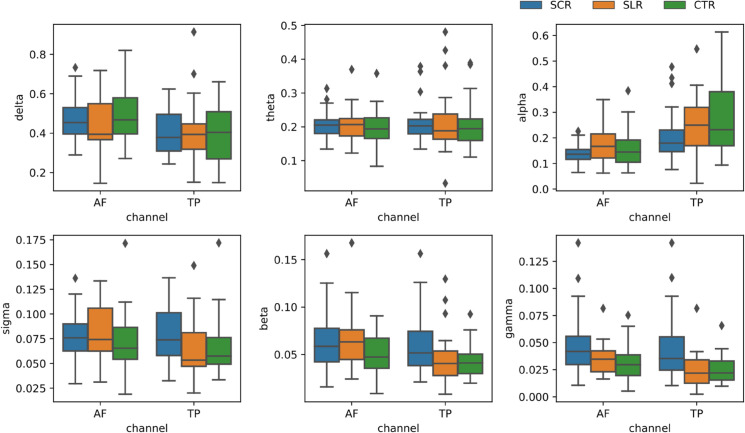
Box plot of the band powers for different groups.

Box plots illustrating the various band powers across different channels for the three groups are presented in Fig 4. Among the six bands (delta, theta, alpha, sigma, beta, and gamma), the sigma, beta, and gamma bands had much lower relative band powers than the delta, theta, and alpha bands. For the relative alpha band power, the median for the SCR group was lower than that of the SLR and CTR groups in both TP and AF channels. Conversely, for theta bands, there was a higher median relative band power for the SCR group compared to the CTR group. In the case of the delta band, in the AF channel, group SLR displayed a substantially lower median band power than others. However, this was not the case in the TP channel.

We performed the Kruskal–Wallis test to determine if the medians for the three groups were significantly different. In the TP channel, the higher frequency (>8 Hz) bands were found to be significantly different: alpha band (*p* = 0.03, $\epsilon^{2}=0.12$, small–medium), sigma band (*p* = 0.01, $\epsilon^{2}=0.18$, medium), beta band (*p* = 0.02, $\epsilon^{2}=0.15$, medium), and gamma band (*p* = 0.0005, $\epsilon^{2}=0.34$, large). For the AF channel, only the gamma band (*p* = 0.009, $\epsilon^{2}=0.20$, medium) was found to be significantly different.

### Effect on persistent homology features

**Fig 5:**
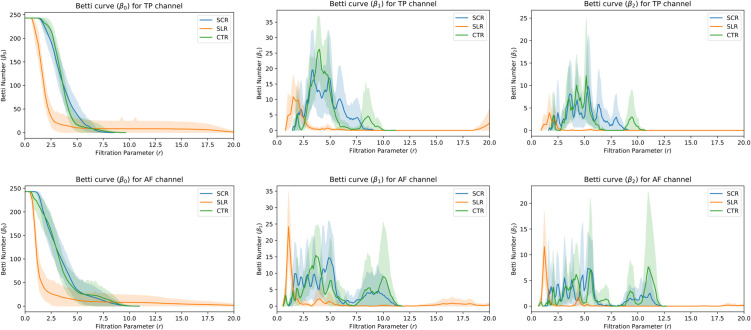
Betti curves averaged across subjects from SCR, SLR, and CTR groups. The top (bottom) panel gives curves for the TP (AF) channel of the EEG. $\beta_{k}$ represents for *k*–th homology group.

- Betti curve β(.): Fig 5 depicts the average Betti curves for the SCR, SLR, and CTR groups across all subjects, categorized by TP (top panel) and AF (bottom panel) channels. The shaded area represents the 95% confidence interval. The Betti numbers, $\beta_{0}$, $\beta_{1}$, and $\beta_{2}$, correspond to the number of connected components, holes, and voids in the data, respectively. For $\beta_{0}$, as *r* increased, the number of connected components decreased. Given the comparable sizes of the point clouds constructed from EEG time series data for all three groups, the $\beta_{0}$ curves showed similar initial values, although their slopes differed. SCR and CTR exhibited similar slopes, while SLR showed distinct slope. Additionally, the peaks of the higher-order Betti curves ($\beta_{1}$ and $\beta_{2}$) revealed distinct patterns across groups and electrode positions (TP/AF).
- Persistence entropy ($S_{k}^{pe}$): Tables 3 and 4 present the mean persistence entropy and the corresponding Kruskal–Wallis *p*-values for each group in the TP and AF channels, respectively. All *p*-values were found to be less than 0.05, demonstrating statistical significance. The SLR group exhibited the highest entropy across all three groups and all homology dimensions in both TP and AF channels. For $S_{0}^{pe}$ and $S_{1}^{pe}$, the values for the SCR and CTR groups were comparable; however, for $S_{2}^{pe}$, SCR showed higher values than CTR in both channels. The corresponding effect sizes were found to be large for all comparisons, indicating substantial group-level differences in entropy across conditions.
- Persistent amplitude (*A*_*k*_): The mean persistence amplitude for each group in the TP and AF channels is also shown in Tables 3 and 4. All *p*-values were found to be less than 0.05, demonstrating statistical significance. Notably, the SLR group exhibited the lowest amplitude across all three groups for all homology dimensions in both the TP and AF channels. For all homology dimensions, the values for the SCR and CTR groups were similar in both channels. The effect sizes for these amplitude-based comparisons were also large in most cases, except for the AF channel where the effect size for *A*_1_ was moderate.

**Table 3:** Results of the Kruskal-Wallis Test on persistent amplitude and persistent entropy measures for TP channel. Effect sizes are reported using epsilon-squared ($\varepsilon^{2}$)

Feature	SCR	SLR	CTR	*p*-value	Effect size ($\varepsilon^{2}$)
*A*_0_	28.32	18.62	28.18	3.0e-03	0.385
*A*_1_	2.10	1.18	2.0	4.5e-03	0.495
*A*_2_	0.89	0.61	0.86	1.8e-03	0.485
$S_{0}^{pe}$	0.80	0.92	0.82	4.4e-09	0.382
$S_{1}^{pe}$	1.39	1.88	1.38	1.9e-09	0.485
$S_{2}^{pe}$	1.89	4.36	1.38	3.0e-09	0.043

**Table 4:** Results of the Kruskal-Wallis Test on persistent amplitude and persistent entropy measures for AF channel. Effect sizes are reported using epsilon-squared ($\varepsilon^{2}$)

Feature	SCR	SLR	CTR	*p*-value	Effect size ($\varepsilon^{2}$)
*A*_0_	26.82	14.70	26.17	2.8e-03	0.338
*A*_1_	1.83	1.19	1.85	4.0e-03	0.307
*A*_2_	0.81	0.50	0.82	1.3e-03	0.233
$S_{0}^{pe}$	0.84	0.96	0.85	3.7e-06	0.338
$S_{1}^{pe}$	1.46	1.91	1.53	2.7e-07	0.300
$S_{2}^{pe}$	2.31	3.49	2.04	5.4e-07	0.220

### Effect on multiscale entropies

**Fig 6:**
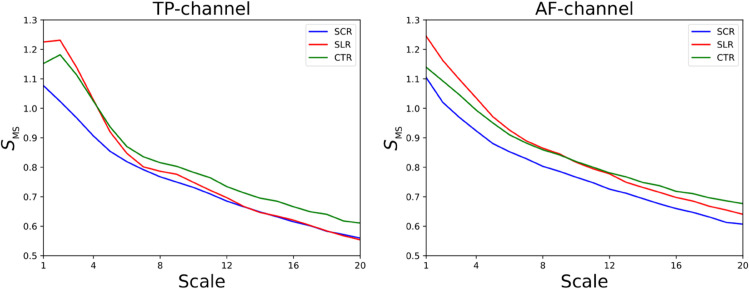
Multiscale entropies for TP and AF channels.

The multiscale entropies *S*_*MS*_ for the three groups in TP and AF channels are depicted in Fig 6. In the TP channel, we noted that at smaller scales, groups SLR and CTR exhibited identical sample entropies but differed from the SCR group. Conversely, at larger scales in the TP channel, groups SCR and SLR displayed identical sample entropies but differed from the CTR group. In the AF channel, a distinct separation was observed between group SCR and the other groups. In the AF channel, groups SLR and CTR were distinguishable either at very small or very large scales.

### Effect on Hodge spectral entropies

**Fig 7:**
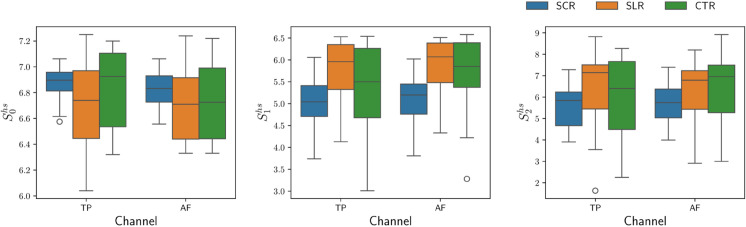
Box plot of the Hodge spectral entropies for different groups. $S_{k}^{hs}$ is the *k*^*th*^ order Hodge spectral entropy associated with the EEG signal.

Box plots depicting the Hodge Spectral Entropies across different channels for the three groups are shown in Fig 7. The labels $S_{0}^{hs},S_{1}^{hs},S_{2}^{hs}$ correspond to the zeroth order, first order, and second order Hodge Spectral entropies. The TP channel showed a consistent upward trend in $S_{1}^{hs}$ and $S_{2}^{hs}$ from the SCR group to the CTR group to the SLR group, in contrast to the opposite trend observed in their corresponding TTB scores. A similar pattern was evident in the AF channel, with the exception that $S_{2}^{hs}$ in group CTR had a slightly higher median compared to group SLR. In the case of $S_{0}^{hs}$, there was an increase from group SCR to group SLR in the TP channel but not in the AF channel.

Additionally, the interquartile range in Fig 7 for group SCR was significantly smaller compared to the SLR and CTR groups across all three orders of entropy. When a Kruskal–Wallis test was performed, we found that the distributions of the three groups were significantly different in the TP channel. We obtained the following *p*-values: *p* = 0.002 for $S_{1}^{hs}$, *p* = 0.03 for $S_{2}^{hs}$, and *p* = 0.3 for $S_{0}^{hs}$. In the AF channel, statistically significant differences were observed for $S_{1}^{hs}$ ($p=8\;\times \;10^{-5}$) and $S_{2}^{hs}$ (*p* = 0.02), but not for $S_{0}^{hs}$ (*p* = 0.2). The corresponding effect sizes ($\epsilon^{2}$) indicated a consistent trend, with $S_{1}^{hs}$ showing the largest effect in both TP ($\epsilon^{2}=0.14$) and AF ($\epsilon^{2}=0.214$) channels, while $S_{0}^{hs}$ yielded negligible effects ($\epsilon^{2}<0.02$) and $S_{2}^{hs}$ reflected small to moderate effects ($\epsilon^{2}=0.06$–0.08). We would like to emphasize that correction methods to control for multiple statistical comparisons were not employed to avoid the risk of inflated type I errors based on the recommendations for pilot studies [54].

### Relation between the Hodge spectral entropy and Total Time Binding (TTB)

We found that the TTB was negatively correlated with second-order Hodge spectral entropy in TP and AF channels for all groups, as indicated in Table 5. All these correlations were significant (*p* < 0.05) across all groups. In the AF channels, while the correlations were negative for all three groups, only the SCR and SLR groups exhibited significance, not the CTR group. Interestingly, action binding and tone binding did not demonstrate such correlational effects. Additionally, the zeroth and first-order Hodge spectral entropy did not exhibit similar effects.

**Table 5:** Pearson correlations (*p*-values) between total time binding (TTB) and second-order Hodge spectral entropy $\left(S_{2}^{hs}\right)$. Bold represents significant results (*p* < 0.05)

Group	TP	AF
SCR	**–0.49 (0.005)**	**–0.42 (0.017)**
SLR	**–0.51 (0.012)**	**–0.56 (0.004)**
CTR	**–0.62 (0.001)**	–0.37 (0.065)

## Discussion

The primary objective of this study was to test whether different forms of self-reflection - self-centered reflection (SCR), selfless reflection (SLR), and control reflection (CTR)—influence the implicit sense of agency, and to identify the corresponding neural correlates. Consistent with our primary hypothesis, intentional time binding (TTB) differed significantly across groups, with SCR increasing and SLR decreasing the binding effect relative to CTR. These results indicate that the orientation of self-reflection dynamically alters the perceived coupling between actions and their outcomes. In line with our secondary hypothesis, distinct EEG signatures were observed across the reflection conditions, both in spectral band powers and in topological features. Furthermore, as hypothesized, Hodge spectral entropy was found to significantly correlate with TTB, suggesting a mechanistic link between topological neural features and the modulation of agency.

To explore how self-reflection alters the implicit sense of agency we have performed the intentional time-binding task post two kinds of self-reflective states, namely self-centered and selfless. To understand the complex interplay between neural activity and sense of agency, we examined various EEG features, including band powers and TDA-based metrics such as Betti curves, persistent entropy, persistent amplitude, and Hodge spectral entropy. Some of the key findings from this study were as follows:

- Total time binding (TTB), an implicit measure of the sense of agency, diminished for the group engaged in selfless reflection (SLR) compared to the control group (CTR). Conversely, compared to CTR, it increased for a group engaged in self-centered reflection (SCR) when considering retrospective and prospective life successes and failures. The result indicated that self-centered (selfless) reflection strengthens (weakens) the associative relationship between an action and its consequence as the doer.
- The Kruskal-Wallis test showed significant differences in the higher frequency bands between the three groups. In the TP channel, alpha, sigma, beta, and gamma bands had statistically significant differences, while in the AF channel, only the gamma band showed a significant difference.
- The distributions of the three groups differed significantly for persistence entropy and amplitude across up to second homology dimensions in both the TP and AF channels. Additionally, Hodge spectral entropy showed significant differences for the first and second homology dimensions in both channels.
- The second-order Hodge spectral entropy of EEG for the TP channels demonstrated a significant negative correlation with TTB for all three groups, indicating that the higher information flow between the higher and lower-order simplicial chains corresponded to a reduced intentional time-binding effect. Higher information flow may be interpreted as more pathways or connections through which topological information can flow or propagate within the simplicial complex and correlated with reducing the binding of actions and their temporal consequences.

Our first major finding was related to the effects of the self-reflective states on sense of agency. While various intentional time-binding tasks have been used to investigate the specific causes of the sense of agency, our experiment employed a canonical intentional binding paradigm, in which all actions produced specific outcomes after a consistent short time interval during the agency blocks. The sense of agency is typically explained through mechanisms such as the brain’s predictive processes [55,56], causal mechanisms [57], and postdictive mechanisms [55], which suggest that the brain rewrites history to strengthen the feeling of agency. The group receiving the SCR intervention showed a greater intentional time-binding effect than the CTR group. As we prompted the subjects to reflect on their past and future successes and failures in the SCR intervention, we likely fostered a sense of self-centeredness. Dambrun et al. [27] stated that overemphasis on the self arises primarily from self-centeredness, indicating a heightened sense that one’s own situation was more significant than others. In contrast, during the CTR or SLR conditions, the self-agency system may not have been as actively engaged, suggesting a potential link to the enhanced time-binding effects in the SCR group. Here, the self-agency system may have caused participants to feel they had caused the outcome, effectively contracting the time interval between action and result.

Recent theoretical advances by Silvanto and Nagai [26] provide a compelling neurocognitive framework relevant to our findings. They propose that vivid mental imagery and a robust sense of agency arise from the integration of interoceptive bodily signals with sensory and cognitive representations, mediated by the insula and anterior cingulate cortex. Within this framework, SCR likely engages this integrative network more strongly, anchoring mental imagery in bodily signals and thereby enhancing the implicit sense of agency measured by intentional time binding. Conversely, SLR condition may shift attention away from interoceptive bodily awareness, disrupting this integrative process and leading to a diminished sense of agency. Thus, Silvanto and Nagai’s model offers a neurobiological explanation for the differential effects of SCR and SLR on agency observed in our study. EEG studies of self-referential processing often focused on brain regions that overlap with the default mode network, especially the medial prefrontal cortex (MPFC) [58–60]. These areas were associated with self-awareness, mind-wandering, and social cognition. The MPFC’s influence on EEG activity in AF7 and AF8 was significant due to their proximity and neural connections. Self-referential processing was commonly examined in relation to the P300 event-related potential component, which reflected the brain’s ability to distinguish between self-related and other-related information. However, there are relatively few studies investigating the EEG topological features associated with self-reflective states. Alpha oscillations were prominently linked to spontaneous self-referential mentation, such as mind-wandering and internal thought processes. They tended to be involved when the brain was at rest, particularly during introspective or self-related thinking. Also, beta and gamma oscillations played important roles in various aspects of self-referential processes [58]. As observed in our case, a statistically significant difference in relative band powers for >8 Hz was quite in line with this. We observed significant differences in topological features across various reflection groups, replicating findings of our earlier work [30]. Consistent with the previous results, self-centered reflections led to lower first- and second-order Hodge spectral entropy than the CTR group (see Fig. 7).

Entropies have been used to study the effects of meditation [61–63]. It was suggested that low complexity in the brain corresponded to a limited range of possible configurations or a narrower scope of conscious experiences [61,64]. Conversely, high-entropy states were believed to encompass a wealth of phenomenological content, indicating more diverse and abundant conscious experiences [65,66]. The third significant finding related to Hodge spectral entropies suggested that, regardless of the intervention, second-order Hodge spectral entropy $\left(S_{2}^{hs}\right)$ was negatively correlated with TTB (see Table 5). This implied that the enhanced phenomenological content of one’s experiences made one less prone to making judgment errors. Conversely, lower Hodge spectral entropy, due to a narrower scope of consciousness resulting from self-centeredness, caused one to make more judgment errors. This study provided initial evidence that topological features could be biomarkers for different cognitive states.

The multiscale entropy results (see Fig 6) suggested that distinctions between SCR and SLR interventions were observed at lower scales in temporoparietal brain regions. This indicated that the self-modulation due to SCR and SLR interventions could be observed at more granular timescales. Conversely, in the anteriofrontal regions, modulations were observed at coarser timescales, indicating that the effects of these interventions might have operated at different timescales in various brain regions.

We acknowledge a few limitations of this study. This study was not pre-registered because at the time of study initiation, there were no clear requirements mandating pre-registration for pilot studies. The primary objective of this pilot study was to generate hypotheses for future confirmatory research. All study methods, hypotheses, and analyses were developed prior to data analysis, and we have reported them transparently to maintain the integrity and reproducibility of our findings. Additionally, given the pilot nature of this study, we did not control for multiple statistical comparisons thereby avoiding the risk of inflated type I errors, as recommended by Moore et al. [54]. As with many feasibility-oriented EEG studies, we prioritized a streamlined setup to enable rapid data collection, and thus, our study employed only four EEG channels, limiting the scope of brain areas under investigation. A longitudinal study incorporating additional sessions and a high-density EEG system could reveal clearer effects. Furthermore, while we utilized a standard intentional time-binding protocol, a more diversified set of experimental variants is warranted to delve deeper into the mechanisms underlying the sense of agency. Though the guided reflection prompts were adapted from previous studies and piloted, future studies should validate these prompts to evaluate the effects of different types of reflections on sense of agencies. The short duration of reflection tasks and reliance on predefined self-reflective exercises might not fully capture the complexity of personal reflection processes. Expanding the scope of study with personalized tasks and integrating detailed psychological and behavioral assessments would offer a more nuanced view of self-reflection. Despite these limitations, our findings provide novel evidence that self-reflection modulates both behavioral and neural markers of the sense of agency, and that topological EEG features such as Hodge spectral entropy can serve as promising biomarkers for capturing these effects.

## Conclusion

In conclusion, our study examined the impact of self-centered versus selfless reflections on the implicit sense of agency, measured by the compression of perceived time between voluntary actions and sensory outcomes. We found that self-centered reflections enhanced the associative binding between actions and consequences, increasing the sense of agency, while selfless reflections had the opposite effect. The study also explored the neural processes underlying selfless and self-centered reflections.

We found statistically significant differences among the self-reflective EEG states in relative band powers and topological neural markers such as persistent amplitude, persistent entropy, and Hodge spectral entropy. Our analysis of Hodge spectral entropy features revealed a significant negative correlation with the sense of agency. This negative correlation implied that higher entropy and increased topological complexity during self-reflective states were associated with lesser judgment errors in perceiving actions or consequences. Consequently, enhanced spectral information in the selfless reflection group was linked with a lesser sense of agency. The situation was exactly the opposite for the self-centered reflection group.

Thus, this study suggests that various self-reflective practices modulated the sense of agency, and the corresponding neural processes were characterized by rich topological signatures. We suggest that topological features like Hodge spectral entropy may serve as a valuable biomarker for different self-reflective and meditative states, acknowledging that interpretations can vary based on context and cognitive tasks. Our future work aims to provide further insights into the topological characteristics of the neural processes behind such cognitive states.
